# Consumer Perception of Food Safety and Valuation of Statistical Life: A Contingent Valuation Study

**DOI:** 10.3390/foods13162597

**Published:** 2024-08-20

**Authors:** Bingjie Liu, Yinuo Na, Yi Li, Dan Wang, Xin Zhang

**Affiliations:** Department of Health Economics, Harbin Medical University, Harbin 150000, China; liu687196@126.com (B.L.); naynuo@126.com (Y.N.); ly897133229@163.com (Y.L.); wd19990612@163.com (D.W.)

**Keywords:** value of statistical life, willingness to pay, food safety, contingent valuation

## Abstract

The value of statistical life (VSL) reflects the trade-off between money and the risk of death. It is a key indicator for conducting regulatory impact assessments. The main purpose of this study was to estimate the VSL in the field of food safety. At the same time, it investigates respondents’ willingness to pay (WTP) for reducing the risk of death from foodborne illnesses and explores the impact of factors such as the average household monthly income, health status, and education level on WTP. We conducted a survey using an open-ended contingent valuation method among 1307 respondents aged 18 and above to determine their WTP. Based on the WTP survey results, we calculated the VSL in the food safety sector. We used binary logit and Tobit models to analyze the influencing factors. The results of the study show that the median WTP for reducing the risk of foodborne illness is CNY 100 (USD 16), and the estimated VSL is approximately CNY 9.09 million (USD 141 million). Indicators such as the average household monthly income, health status, and education level are important factors affecting WTP. This study will help practitioners, researchers, and policymakers understand the current population’s attitudes towards food safety regulations and determine the priorities for regulatory implementation. Future research can explore the effects of different elicitation methods, cultural differences, and regional variations on WTP and VSL.

## 1. Introduction

Foodborne illnesses are infections or toxic diseases caused by bacteria, viruses, parasites, or chemicals contaminating food or water that enter the human body. The World Health Organization (WHO) estimates that food safety issues annually lead to approximately 600 million illnesses and 420,000 deaths globally [1]. In low- and middle-income countries, foodborne diseases result in an annual productivity loss of USD 95.2 billion [2]. Currently, there is limited research on the burden of foodborne diseases in China, and nationwide studies are lacking. However, a study has used models to estimate that bacterial foodborne diseases in China result in 94.117 million cases annually, leading to 8530 deaths, with a case fatality rate of 0.0091% [3].

Being a crucial factor affecting human health [1], governments worldwide prioritize food safety as a key public health initiative [4]. They implement scientifically sound interventions to regulate the food industry, aiming to reduce food safety risks and safeguard public health. The “Healthy China 2030” blueprint also proposes strengthening food safety regulation through multiple approaches, including the food safety standards system, risk monitoring, and risk assessment, to ensure food safety [5]. In situations with limited resources, policymakers need to quantify the costs and benefits of regulations pertaining to health risk reduction in order to prioritize their implementation. Risk reduction measures are the main income of food safety regulations, but are difficult to measure due to a lack of market prices [6]. At this point, the concept of “the value of a statistical life” needs to be introduced to quantify the health benefits of regulatory measures.

VSL is the cost that people are willing to pay (WTP) to reduce the risk of death or the risk of death that increases their willingness to accept compensation. Commonly used in cost–benefit analyses of health and safety policies and regulations, it defines the monetary value of risk reduction. VSL does not refer to a particular value of life, but reflects people’s valuation of the measure between risk and money [7]. It refers to the additional costs people are willing to incur to reduce health risks, which is the marginal rate of substitution between income and the risk of death.

VSL has been extensively researched and applied in regulatory assessments in developed countries. While developing countries have begun estimating local VSL values, particularly in environmental, health, and transportation sectors, there has been relatively limited research on VSL in the field of food safety. Sweden obtained a VSL figure in the field of food safety by surveying citizens on WTP for reducing the risk of salmonella in chicken meat [8]. The United States Department of Agriculture used a VSL of USD 9.7 million (2018 figure) when estimating productivity losses due to foodborne illnesses [9].

Existing studies have shown that VSL is highly sensitive to the estimation methods used in different industries of research, population characteristics, and economic development. It is not only impacted by socioeconomic development but also significantly affected by different research fields. According to Keller et al.’s [10] review, VSL in the health sector, labor market, and road transportation industry were USD 6.8 m, USD 8.7 m, and USD 5.3 m, respectively (2019 figures). Therefore, it is very important to estimate VSL in different industries and countries [11,12].

Based on the above premises, the objectives of this study comprised: (1) obtaining people’s WTP for reducing the risk of death from foodborne diseases; (2) exploring the factors influencing WTP; and (3) estimating the VSL in the field of food safety based on WTP survey results. This study aims to assist relevant practitioners in understanding the public’s acceptance of paying for food safety regulations and their WTP, identifying the key factors influencing WTP. It also provides assistance to policymakers in conducting food safety regulation assessments.

## 2. Literature Review

### 2.1. Willingness to Pay for Food Safety

Existing research indicates that consumers’ WTP is influenced by various factors, including personal characteristics, risk perception, economic conditions, and awareness of food safety and certification. These factors interact with one another to collectively determine consumers’ WTP for safe food. Research results worldwide show that in both developed and developing countries, consumers’ WTP is significantly influenced by similar factors.

Multiple studies have found that consumers’ personal characteristics significantly impact their WTP. Researchers both domestically and internationally have empirically verified the effects of factors such as education level, income, gender, and age on consumers’ WTP [13,14]. However, the influence of these factors on WTP is not consistent across different literature. Consumers’ perception of food safety risks significantly affects their WTP, with those possessing a stronger ability to perceive risks being more willing to pay for food safety [15,16]. Household income level and payment capability are also important economic factors. Most research findings indicate that an increase in income has a positive effect on consumers’ WTP [17,18,19]. Additionally, consumers’ awareness of food safety and certification significantly impacts their WTP. Studies by Guo [20] and Yin [21] have shown that consumers’ subjective knowledge, trust in the government, and understanding of safety certifications significantly influence their WTP. Haghjou [22] also found that income, environmental concern, and awareness of organic food affect Iranian consumers’ WTP.

### 2.2. The Value of a Statistical Life in Food Safety

Since the 1970s, VSL has been extensively studied and applied in regulatory assessments in developed countries. Although many developing countries have begun estimating local VSL in recent years, the focus has primarily been on environmental, health, and transportation sectors. There are relatively few studies on VSL in the field of food safety. China began studying the value of life in the 1990s, with research primarily focused on occupational safety. There has yet to be research on the statistical value of life in the field of food safety.

In 2009, Swedish scholar Sundström [8] applied the contingent valuation method (CVM) to survey citizens’ WTP for reducing the risk of Salmonella in chicken, resulting in a VSL of SEK 13.3 million (USD 1.23 million) in the field of food safety. Another study conducted in Sweden used a discrete choice experiment (DCE) to estimate the VSL related to reduced mortality risk from Campylobacter, which was calculated to be USD 695,000,000 [23]. Haninger et al. employed the CVM to study foodborne risks associated with acute illnesses, estimating the VSL for adults in the United States to be between USD 7,200,000 and USD 51,200,000 [24]. Additionally, research using the DCE to assess the statistical value of life related to health risks from drinking water found VSL estimates ranging from USD 1,194,693 to USD 15,212,425 [25]. There are significant variations in VSL values across different studies.

Existing research primarily employs the CVM and the DCE. The application of these methods in different countries and regions has revealed significant variations in VSL, reflecting differences in value perception across different socioeconomic contexts. Among these methods, the CVM is currently the most widely used for studying the statistical value of life. Historically, this method has been primarily applied to evaluate environmental and resource values. But in recent years, it has become an important reference for measuring the value of food safety. However, given the substantial differences in market environments and consumer characteristics between China and international contexts, the results of related studies have limited applicability for Chinese producers and policymakers.

## 3. Study Design

### 3.1. Stated Preference and Open-Ended Contingent Valuation

Methods for eliciting VSL can be divided into two categories: revealed preference (RP) and stated preference (SP). RP relies on real market data and analyzes individual market behaviors to estimate VSL. SP methods, on the other hand, are based on hypothetical scenarios and use surveys to elicit individuals’ WTP, making it possible to estimate VSL in the absence of market data. Compared to RP, SP is more flexible. Because scenarios are hypothetical, the SP can capture preferences in a wider range of situations, whereas RP, which relies on market data, reflects preferences in specific market contexts. Based on the aforementioned advantages, SP has been widely used to assess public goods or private non-market goods [10].

The CVM is a typical SP estimation approach. This method involves directly asking individuals in a hypothetical market setting about their WTP for reducing the risk of death. The guiding technique is the key to conduct CVM research, which directly affects the validity and reliability of the results [26]. These methods include the bidding game, payment card, binary choice, and open-ended formats. Our study employed an open-ended elicitation technique to conduct a WTP questionnaire survey. This elicitation method does not provide respondents with any bidding value information and directly asks them about their maximum WTP. The open-ended elicitation technique is advantageous for its simplicity, directness, and the absence of starting-point bias [27].

### 3.2. Data Collection

Our study targeted individuals aged 18 and above in China as respondents. The primary reasons for selecting this demographic included the following. First, younger respondents may find it difficult to make independent food consumption decisions [28]. Second, the questionnaire employs an open-ended elicitation method, which requires consideration of the respondents’ ability to understand the questions. Based on a combination of China’s 2020 GDP and geographical distribution, six provinces/municipalities (Guangdong, Hebei, Heilongjiang, Hunan, Shanghai, and Sichuan) were selected for the WTP survey. The formal survey commenced in July 2021, using the online survey platform Wenjuanxing to distribute the questionnaire. Wenjuanxing is a professional online survey company in China, and previous studies have evaluated its suitability for obtaining national samples at a low cost [29]. After collecting the online questionnaires, two researchers independently checked, organized, and coded the responses. In cases of disagreement, a third researcher acted as an arbitrator. To control the quality of the questionnaires, those completed in an excessively short amount of time were deleted. Logical checks were performed on the questionnaires to exclude any responses that were clearly inconsistent with the questions, ensuring the rigor of the empirical data and the reliability of the conclusions.

### 3.3. Questionnaire Design

A WTP for food safety survey questionnaire was designed using an open-ended elicitation method. The first page of the questionnaire clearly stated that this was an anonymous survey conducted by Harbin Medical University aimed at investigating people’s WTP for reducing the risk of death from foodborne diseases. Additionally, we informed respondents that the survey was solely for research purposes and that their responses would be kept strictly confidential. If respondents agreed, they could then begin filling out the questionnaire.

**Figure 1:**
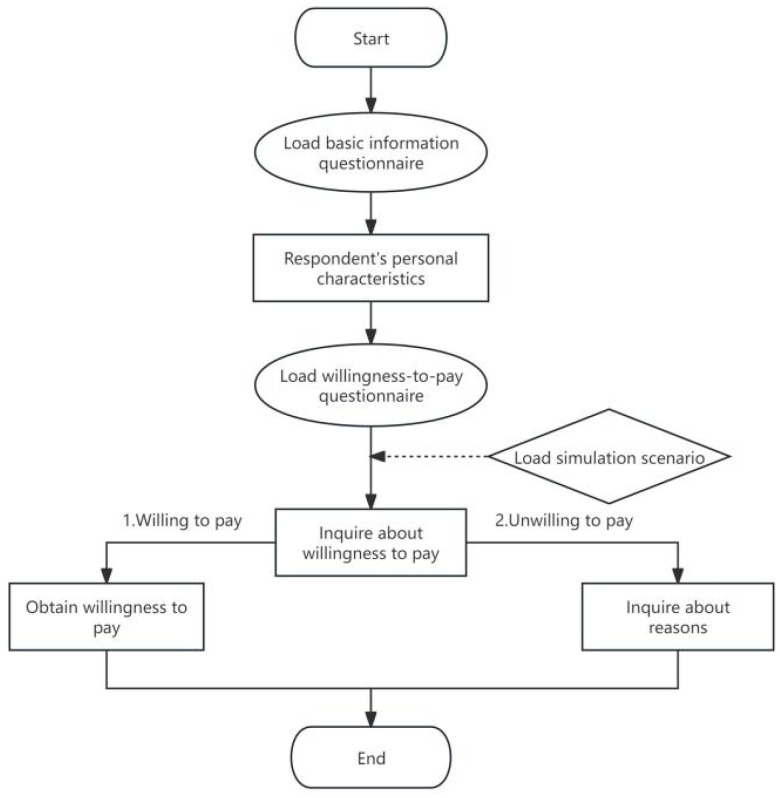
Questionnaire logic diagram.

The questionnaire comprised two parts. The first part of the survey collected basic information of respondents, including gender, age, education, marriage, employment, family income, and other personal and family characteristics. The second part collected respondents’ WTP for implementing stricter food safety standards (that could reduce the risk of foodborne disease-related mortality). Respondents were initially asked, “Are you willing to pay for stricter food safety standards?” If the answer was yes, they were asked, “How much are you willing to additionally pay?” If they answered no, they were asked to furnish the reasons for their disagreement. The selected independent variables included sociodemographic information such as sex, age, and income. Province was used as a control variable, as shown in Table 1. The questionnaire logic is illustrated in Figure 1.

To help respondents understand the survey background, the questionnaire provided a description of the definition and mortality data of global foodborne diseases: “Foodborne illnesses are infections or toxic diseases caused by bacteria, viruses, parasites, or chemicals contaminating food or water that enter the human body” [1]. According to the relevant statistics, the mortality risk from foodborne diseases in China in 2015 was 1.42 per 100,000. We presented a scenario in which the government implemented strict food safety standards. Implementation of these standards could lead to improvements in food safety, reducing the mortality risk from eating unsafe food by two-thirds (approximately one death per 100,000 people. We developed a purchasing power illustration table based on the current year’s market price to show the purchasing power of the Chinese yuan which helped respondents determine their WTP and improve the validity of our survey (Table 1). To ensure the quality and reliability of the data, three key measures were implemented in this study: checking the logic of the questionnaire to ensure the rationality of the question setting, removing questionnaires with short completion times to improve the accuracy of the data, and identifying and eliminating questionnaires with logic contradictions.

The independent variables included sex, age, and employment status, as shown in Table 2.

### 3.4. Model Design

#### 3.4.1. Measurement Method of Statistical Life Value

Referring to Andersson’s monograph published in 2020, VSL is the marginal rate of substitution between mortality risk and wealth. Its theoretical model is a utility framework based on individual survival conditions, in which individuals pursue utility maximization:

(1) $$EU\left(w,p\right)=pu_{a}\left(w\right)+\left(1-p\right)u_{d}\left(w\right)$$

where p is the individual survival probability, w is the wealth owned by individuals. u_a_(w) and u_d_(w) represent the utility of wealth in the states of individual survival and mortality, respectively. The model assumes that both utility and marginal utility are larger if the individual is alive.

Assuming C(ε) is the WTP for the reduction in mortality risk ε, substitute it into Equation (1).

(2) $$\left(p+w\right)u_{a}\left(w-C\left(\varepsilon \right)\right)+\left(1-p-\varepsilon \right)u_{d}\left(w-C\left(w\right)\right)=EU_{0}$$

VSL can then be calculated by taking the limit of WTP as ε ≌ 0

(3) $$VSL=-\frac{dw}{dp}{|}_{EU\;constant}=\frac{u_{a}\left(w\right)-u_{d}\left(w\right)}{p{u^{\prime }}_{a}(w)+(1-p){u^{\prime }}_{d}\left(w\right)}$$

However, in empirical studies, the reduction in the mortality risk is usually limited. Researchers will ask the respondent about the WTP with a small (Δp) change in risk, and the VSL will be obtained by the ratio of an individual’s wealth change (such as WTP) to the risk change [30].

(4) $$VSL=\frac{WTP}{\Delta p}$$

#### 3.4.2. Model Construction

Stata was used to construct binary logit and Tobit models to analyze respondents’ WTP for food safety. While estimating VSL, we also studied the impact of different factors on respondents’ WTP.

According to the basic idea of the logit model, the dependent variable $y_{i}$ is respondent i(i = 1,…, n) WTP additional payments for safe food, with a value of 1 indicating yes and 0 indicating no. $x_{ij}$(i = 1,…n; j = 1,…, m) represents possible factors such as the average monthly household income level and education level, etc. of respondent i. $B_{j}$(j = 1,…, m) is the regression coefficient, $\beta_{0}$ is the constant term, p is the probability of the dependent variable taking the value of 1, $\varepsilon_{i}$ is the random error, and our binary logit model is obtained [31].

(5) $$\ln\left(\frac{p_{i}}{1-p_{i}}\right)=\beta_{0}+\sum_{j-1}^{m}\beta_{j}x_{ij}+\varepsilon_{i}$$

Because the payment level of respondents obtained by the questionnaire was limited on the left side (i.e., payment level ≥ 0), the Tobit model was used to estimate the impact of explanatory variables on the payment level of respondents:

(6) $$\begin{array}{l} y_{i}*=\beta X_{i}+\varepsilon_{i},\varepsilon_{i}\sim N(0,\sigma^{2})\\ y_{i}=y_{i}*,\;\mathrm{if}\;y_{i}*>0\\ y_{i}=0,\;\mathrm{if}\;y_{i}*\leq 0 \end{array}$$

where y* is the latent variable observed when the latent variable is >0; $y_{i}$ is the dependent variable, which is the payment level of the respondents; $X_{i}$ is a series of explanatory variables such as gender, age, and average monthly household income level; β is the coefficient of variable estimation; and $y_{i}$ is a random disturbance term following a normal distribution with a mean of 0 and a variance of $\sigma^{2}$ [32].

### 3.5. Research Hypothesis

Current research findings indicate that income has a significant positive impact. At the same time, income consumption theory suggests that higher income generally leads to an increased propensity to consume. Therefore, this study proposes Hypothesis 1: Income has a significant positive effect on both the respondents’ WTP and the price they are willing to pay. Some studies suggest that individuals with higher education levels have stronger awareness of food safety and a greater perception of food safety risks [33,34,35]. Thus, this study proposes Hypothesis 2: Education level has a significant positive effect on both the respondents’ WTP and the price they are willing to pay. Respondents with higher food expenditures may be more concerned about food safety issues and therefore have a stronger WTP [33,36]. Hence, this study proposes Hypothesis 3: Average monthly food expenditures have a significant positive effect on both the respondents’ WTP and the price they are willing to pay. Health may have a positive impact on respondents’ WTP [37]; therefore, this study proposes Hypothesis 4: Health has a significant positive effect on both the respondents’ WTP and the price they are willing to pay.

## 4. Results and Discussion

### 4.1. Sample Characteristics

A total of 1307 questionnaires were collected in this study, and 1259 were retained after screening. The distribution was as follows: Heilongjiang 191, Hebei 202, Hunan 198, Sichuan 206, Shanghai 227, and Guangzhou 235. The gender ratio of respondents was balanced, with 651 males (51.71%) and 608 females (48.29%). The majority of respondents were urban residents, accounting for 68.15% of the total. The respondents were mainly young and middle-aged, with an average age of 31 years (range, 18–45 years). Most respondents had a high level of education, which made them more likely to understand the open-ended questionnaire. Of the respondents, 61.95% were married and 61.56% had children under the age of 15. The respondents’ self-rated health status was relatively good. Of the total respondents, 49.72% had an average income of CNY 5000–15,000. Table 3 presents the descriptive statistics for respondents’ characteristics.

### 4.2. Calculation of the Value of Life

This study used respondents’ WTP to estimate VSL. The WTP for food safety proved to be left-skewed; therefore, it was prudent to choose the median value to calculate VSL. The results show that Chinese consumers’ median WTP for stricter food safety standards is CNY 100 (USD 16). When the WTP was substituted in Equation (4), the VSL was CNY 9,090,121.32 (USD 1,408,993.46). As shown in Table 4.

Although the contingent valuation method (CVM) is widely used, it still faces several challenges that remain unaddressed. Therefore, this study employed multiple strategies in the questionnaire design to minimize potential issues associated with the CVM. First, stated preference (SP) scenarios in CVM surveys make it difficult for respondents to fully consider their budget constraints, often resulting in higher VSL estimates compared with revealed preference (RP) methods [38]. To address this issue, we prompted the respondents via the questionnaire to consider the purchasing power of the amount they were willing to pay, ensuring the authenticity of their WTP. Second, when dealing with unfamiliar fields, such as food safety, it is crucial to facilitate respondents’ understanding of the key points of SP surveys. Therefore, researchers must consider scenarios that are meaningful and easily understandable [39]. In our questionnaire, we provided detailed descriptions of the current burden of foodborne diseases in China and succinctly outlined hypothetical scenarios. Additionally, the study focused largely on young and highly educated urban residents, who were more adept at accepting and comprehending new information. This group exhibited a strong ability to accept and understand new information, which helped them grasp the content of the questionnaire better, thus improving the validity of the survey.

The VSL obtained in this study is significantly lower than the median global VSL estimate of CNY 39.32 million (USD 5.70 million) reported in Keller’s 2021 [10] study. Currently, there is a lack of research on VSL in the field of food safety in China. Compared to studies in other fields within China, the VSL estimated in this study was relatively high. For example, Yu Hao et al. had found that the VSL for air pollution in China, based on residents’ WTP to reduce its health impacts, was CNY 1.53 million yuan [40]. Another study conducted in Beijing estimated the VSL for air pollution to be 5.54 million yuan [41]. Research focusing on traffic safety in Hangzhou indicated a VSL of 3.87 million yuan for drivers and 3.36 million yuan for non-drivers [42]. In the field of occupational safety, Zhang Yu et al. estimated the VSL for construction workers in Chengdu as approximately 4.29 million yuan [43]. Compared to the VSL estimated in the field of food safety in other countries, the VSL in our study is also relatively low [8,23,24].

Some studies opined [7,44,45] that individuals tend to overestimate the smaller risks of mortality while underestimating the larger risks. Our study used a WHO-estimated mortality rate of 1.42 per 100,000 for foodborne diseases to calculate VSL, resulting in a significantly lower mortality risk compared to other fields in which VSL studies had been conducted. Therefore, the relatively high VSL values found in this study may be attributed to the perception that a lower mortality risk influences individuals’ WTP. Furthermore, current public concern regarding food safety in China surpasses that of environmental, traffic, and occupational safety issues. According to surveys on public security perceptions and feelings in China [46], food and drug safety (38.45%) ranks fourth among “the most concerning security issues for respondents,” surpassing concerns about ecological environment safety (35.36%) and traffic safety (31.82%). This indicates that food safety is attracting widespread attention in China. This heightened concern is likely to enhance people’s awareness of the importance of food safety, thereby increasing their WTP for quality control and safety measures in this domain.

### 4.3. Reasons for Respondents’ Refusal to Pay

**Figure 2:**
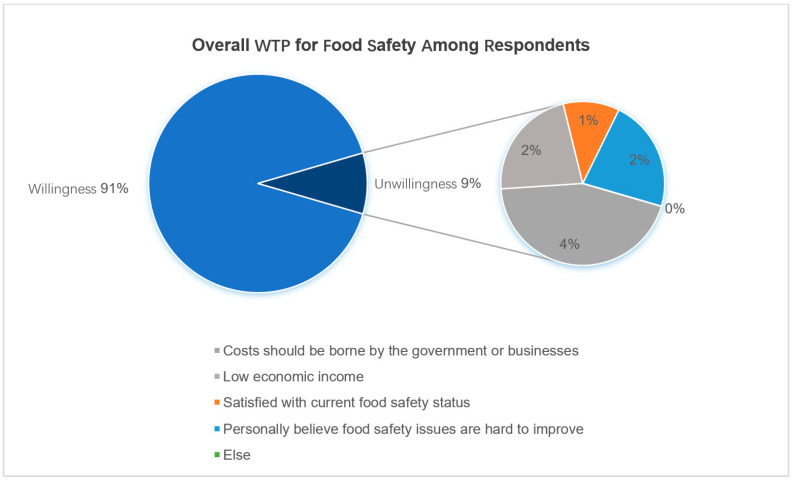
Statistical life value of food safety.

A total of 105 people were unwilling to make additional payments for stricter food safety standards, accounting for 8.3% of the respondents. As shown in Figure 2, “The expense should be covered by the government/company” was the primary reason for respondents’ refusal to pay more, followed by “Belief that food safety issues are difficult to improve.” “Low economic income,” and “Satisfaction with the current food safety situation.” If “Low economic income” in considered as “Real zero payments” and other reasons as “Protest zero payment,” it amounts to a protest rate of 6.59%. A low protest rate in the questionnaire indicates a well-designed survey.

### 4.4. The Factors of WTP

The logit regression results indicated that education, marital status, and health status had a significant impact on respondents’ WTP. Education was an important factor affecting consumers’ WTP, with higher levels of education correlating with a stronger WTP for food safety. Marriage had a positive impact on respondents’ WTP choice–married respondents were more WTP for food safety than others were. Healthy respondents were likewise willing to pay extra for increased peace of mind about food safety.

The Tobit model regression results indicated education, household income, food expenditure, and health status as having significant effects on consumers’ inclination toward making additional payments in the interests of food safety. Specifically, education and health status positively influenced increased payments, which is consistent with their effects on WTP. Household income significantly affects the additional payments that consumers are willing to pay, with both the model coefficients and marginal effects showing an increasing trend, indicating that higher-income households are willing to pay more for safe foods. Food expenditure also positively impacts consumers’ additional payments; respondents with food expenditure below 1000 yuan would only agree to the lowest costs, whereas those above 3000 yuan were willing to pay the highest increases in costs. Table 5 presents the regression results for the binary logit and Tobit models.

Education is an important factor influencing WTP and VSL. Some studies have found a positive correlation between education and WTP, indicating that individuals with higher education levels are more WTP for risk reduction [47]. However, other studies have reported negative or even no correlation between education level and WTP [42,48]. The results of our study show that education is a significant factor affecting respondents’ WTP for food safety, with higher-educated respondents demonstrating a stronger WTP, thus supporting research hypothesis 2. This may be because individuals with higher education levels have a better understanding of the impact of food safety on health, leading to a greater WTP. The impact of health on VSL is uncertain [49]; however, in our study, health has a significant positive effect on both respondents’ WTP and additional payments, supporting research hypothesis 4. This partially supports hypotheses 1 and 3. Average Household monthly income and average monthly food expenditure only had a significant positive impact on additional payments but did not significantly increase the probability of their willingness to pay. This may be because, when respondents decide whether to pay, they consider factors beyond income and food expenditure, such as food safety awareness, health status, and personal preferences, which may prevent income and food expenditure from showing significant effects on willingness to pay. In contrast, when determining the specific price for additional payments, the direct impact of income and food expenditure on payment capacity becomes more apparent, thus showing a significant positive relationship [50,51]. It is generally believed that VSL increases with individual income, which aligns with the findings of this study: as average household monthly income increases, respondents’ additional payments also increase. Household monthly food expenditure can reflect respondents’ concern for food safety to some extent, as healthier foods typically come at a higher price. In this study, controlling for household size, household monthly food expenditure positively influenced additional payments.

## 5. Conclusions

Our article applied open-ended CVM to investigate the WTP for food safety among individuals aged 18 and older in six provinces/municipalities in China. The survey obtained the WTP for reducing the risk of death from foodborne diseases and analyzed its influencing factors. More importantly, the WTP survey results were used to estimate the VSL in the field of food safety. The results showed that the median WTP was 100 yuan (USD 16), leading to an estimation of the VSL in the food safety sector in China to be approximately 9.09 million yuan (USD 141 million), which is higher than previous VSL estimates in other risk areas in China. Educational level, marital status, and health status were significant socioeconomic factors influencing consumers’ WTP. Educational level, average monthly household income, average monthly food expenditure, and health status significantly impacted consumers’ additional payments.

Applying the VSL to food safety regulation can provide a quantitative health risk assessment tool for cost–benefit analysis and the evaluation of food safety-related regulations. This is the first attempt to estimate the VSL in the field of food safety in China, and this study provides key parameters for assessing the economic feasibility of food safety regulations, helping the government allocate resources more rationally and enhancing the scientific basis of decision-making. This study surveyed individuals aged 18 and older, but the affected populations of foodborne diseases mainly include vulnerable groups such as children and the elderly, indicating a need for further investigations targeting these groups in the future. Additionally, VSL is influenced by various macro factors such as cultural background, socioeconomic development, and demographic characteristics, resulting in significant differences between countries and regions. Therefore, caution should be exercised when using VSL from other countries or regions.

This study has certain limitations. First, contingent valuation is an SP method, and surveys conducted based on hypothetical scenarios may not fully reflect real-world situations. Second, another challenge faced by contingent preference surveys is that respondents who are unfamiliar with the field may exhibit biases in interpreting and understanding the questionnaire. Third, the study mainly focused on young adults aged 18 years and older and lacked sufficient samples from children and older populations.

## Figures and Tables

**Table 1:** Purchasing power of different amounts.

CNY (Yuan)	Purchasing Power
5	Buy a bottle of milk
8	Have a breakfast
10	Take a taxi
20	Drink a cup of milk tea
30	Buy a book
40	Eat KFC once
50	To see a movie
80	Get a shoulder and neck massage
100	Go to an amusement park

**Table 2:** Description of variables.

Variable	Categories
Gender	Female, male
Age	18–35, 36–70
Employment	Unemployed/other, student, employed
Education	Secondary school, high school, bachelor’s degree, master’s degree or above
Marriage	Singe/divorced/widowed, married
Location	Rural, urban
Household income (CNY per month)	Less than 5000, 5000–15,000, more than 15,000
Food expenditure (CNY per month)	Less than 1000, 1000–2000, 2000–3000, more than 3000
Children (age < 15)	No, yes
Health status	Poor, general, good
Province	Shanghai, Sichuan, Guangdong, Hebei, Hunan, Heilongjiang
WTP	Unwillingness, willingness

**Table 3:** Description of basic features.

Variable	Option	Evaluation	Number of Samples	Proportion	Willing to Pay Ratio	Average Amount Paid (CNY)
			(Persons)			
Gender	Female	0	608	48.29	551 (90.63%)	1056.32
	Male	1	651	51.71	599 (92.01%)	1026.98
Age	18–35	0	950	75.46	877 (92.32%)	1094.35
	36–70	1	309	24.54	273 (88.35%)	877.60
Employment	Unemployed/other	0	249	19.78	223 (89.56%)	785.04
	Employed	1	1010	80.22	927 (91.78%)	1104.29
Education	Under bachelor’s degree	0	342	27.16	290 (84.80%)	854.56
	Bachelor’s degree or above	1	917	72.84	860 (93.78%)	1110.74
Marriage	Singe/divorced/widowed	0	479	38.05	429 (89.56%)	934.77
	Married	1	780	61.95	721 (92.44%)	1106.48
Location	Rural	0	401	31.85	367 (91.52%)	814.00
	Urban	1	858	68.15	783 (91.26%)	1147.32
Household income (CNY per month)	Less than 5000	0	233	18.51	196 (84.12%)	684.54
	5000–15,000	1	626	49.72	573 (91.53%)	831.16
	More than 15,000	2	400	31.77	381 (95.25%)	1577.53
Food expenditure (CNY per month)	Less than 1000	0	235	18.67	201 (85.53%)	483.32
	1000–2000	1	372	29.55	341 (91.67%)	866.56
	2000–3000	2	324	25.73	299 (92.28%)	899.65
	More than 3000	3	328	26.05	309 (94.21%)	1778.62
Children (age < 15)	No	0	484	38.44	436 (90.08%)	808.95
	Yes	1	775	61.56	714 (92.13%)	1186.17
Health status	General or poor	0	273	21.68	232 (84.98%)	769.55
	Good	1	986	78.32	918 (93.10%)	1116.35
Province	Shanghai	0	227	18.03	208 (91.63%)	865.41
	Sichuan	1	206	16.36	191 (92.72%)	686.05
	Guangdong	2	235	18.67	223 (94.89%)	1437.42
	Hebei	3	202	16.04	191 (94.55%)	1095.16
	Hunan	4	198	15.73	185 (93.43%)	1457.03
	Heilongjiang	5	191	15.17	152 (79.58%)	657.22

**Table 4:** Statistical life value of food safety.

Willingness to Pay	Willing to Pay	Unwilling to Pay
sample size	1154	105
Mean (yuan)	1041.15	/
Standard deviation (yuan)	3823.48	/
Median (yuan)	100	/
Median VSL (yuan)	9,090,121.32	/

**Table 5:** Binary logit model and Tobit model estimates results.

Variable	Level	Willingness to Pay (Logit Model)	Additional Payments (Tobit Model)	
		Coefficient	Coefficient	Marginal Effect
Gender		0.148	0.155	0.149
Age	Age2	−0.131	−0.206	−0.198
Employment	Employment2	−0.263	−0.066	−0.063
Education	Education2	0.735 ***	0.466 ***	0.445 ***
Marriage	Marriage2	0.615 **	0.258	0.247
Location	Location2	−0.320	0.088	0.084
Household income (CNY per month)	Household income2	0.104	0.385 *	0.366 *
	Household income3	0.636	0.955 ***	0.918 ***
Food expenditure (CNY per month)	Food expenditure2	0.369	0.534 **	0.510 **
	Food expenditure3	0.301	0.463 *	0.442 *
	Food expenditure4	0.384	0.542 **	0.518 **
Children (age < 15)	Children2	−0.375	0.052	0.049
Health status	Health status2	0.810 ***	0.428 **	0.409 **
Province	Province2	0.566	0.703 ***	0.674 ***
	Province3	0.799 **	0.698 ***	0.668 ***
	Province4	0.848 **	0.934 ***	0.899 ***
	Province5	0.577	0.893 ***	0.858 ***
	Province6	−0.568	−0.397	−0.371

Note: ***, **, and * indicate that the variable is significant at the 1%, 5%, and 10% statistical levels, respectively. The values in parentheses represent the reference group.

## Data Availability

The raw data supporting the conclusions of this article will be made available by the authors without undue reservation.
